# Clinical management and diagnosis of CLN2 disease: consensus of the Brazilian experts group

**DOI:** 10.1055/s-0043-1761434

**Published:** 2023-04-14

**Authors:** Leticia Pereira de Brito Sampaio, Maria Luiza Giraldes de Manreza, André Pessoa, Juliana Gurgel-Giannetti, Ana Carolina Coan, Hélio van der Linden Júnior, Emília Katiane Embiruçu, Adélia Maria de Miranda Henriques-Souza, Fernando Kok

**Affiliations:** 1 Universidade de São Paulo, Faculdade de Medicina, Hospital das Clínicas, São Paulo SP, Brazil.; 2 Universidade Estadual do Ceará, Hospital Infantil Albert Sabin, Fortaleza CE, Brazil.; 3 Universidade Federal de Minas Gerais, Faculdade de Medicina, Hospital das Clínicas, Belo Horizonte MG, Brazil.; 4 Universidade Estadual de Campinas, Faculdade de Ciências Médicas, Campinas SP, Brazil.; 5 Instituto de Neurologia de Goiânia, Goiânia GO, Brazil.; 6 Universidade do Estado da Bahia, Hospital Universitário Professor Edgard Santos, Salvador BA, Brazil.; 7 Instituto de Medicina Integral Professor Fernando Figueira, Recife PE, Brazil.

**Keywords:** Neuronal Ceroid-Lipofuscinoses, Language Development Disorders, Consensus, Enzyme Replacement Therapy, Epilepsy, Lipofuscinoses Ceroides Neuronais, Transtornos do Desenvolvimento da Linguagem, Consenso, Terapia de Reposição Enzimática, Epilepsia

## Abstract

Neuronal ceroid lipofuscinosis type 2 (CLN2) is a rare neurodegenerative genetic disease that affects children in early life. Its classic form is rapidly progressive, leading to death within the first 10 years. The urge for earlier diagnosis increases with the availability of enzyme replacement therapy. A panel of nine Brazilian child neurologists combined their expertise in CLN2 with evidence from the medical literature to establish a consensus to manage this disease in Brazil. They voted 92 questions including diagnosis, clinical manifestations, and treatment of the disease, considering the access to healthcare in this country. Clinicians should suspect CLN2 disease in any child, from 2 to 4 years old, with language delay and epilepsy. Even though the classic form is the most prevalent, atypical cases with different phenotypes can be found. Electroencephalogram, magnetic resonance imaging, molecular and biochemical testing are the main tools to investigate and confirm the diagnosis. However, we have limited access to molecular testing in Brazil, and rely on the support from the pharmaceutical industry. The management of CLN2 should involve a multidisciplinary team and focus on the quality of life of patients and on family support. Enzyme replacement therapy with Cerliponase α is an innovative treatment approved in Brazil since 2018; it delays functional decline and provides quality of life. Given the difficulties for the diagnosis and treatment of rare diseases in our public health system, the early diagnosis of CLN2 needs improvement as enzyme replacement therapy is available and modifies the prognosis of patients.

## Resumo

Lipofuscinose ceróide neuronal (CLN2) é uma doença genética neurodegenerativa rara que afeta crianças nos primeiros anos de vida. A sua forma clássica é rapidamente progressiva, levando à morte nos primeiros 10 anos. A necessidade de um diagnóstico precoce aumenta com a disponibilidade do tratamento de terapia enzimática. Um painel de nove neurologistas infantis brasileiros combinou sua experiência em CLN2 com evidências da literatura médica para estabelecer um consenso no manejo desta doença no Brasil. Eles votaram 92 questões abordando diagnóstico, manifestações clínicas e tratamento, considerando o acesso à saúde no Brasil. Deve-se suspeitar de CLN2 em qualquer criança de 2 a 4 anos de idade que apresente atraso de linguagem e epilepsia. Apesar da forma clássica ser a mais prevalente, podem ser encontrados casos atípicos com diferentes fenótipos. Eletroencefalograma, ressonância magnética, testes moleculares e bioquímicos são as principais ferramentas para investigar e confirmar o diagnóstico. No entanto, o acesso aos testes moleculares é limitado no Brasil, necessitando contar com o apoio da indústria farmacêutica. O manejo da CLN2 deve envolver uma equipe multidisciplinar e focar na qualidade de vida dos pacientes e no apoio familiar. A terapia de reposição enzimática com Cerliponase alfa é um tratamento inovador aprovado no Brasil desde 2018; ele retarda o declínio funcional e proporciona qualidade de vida. Diante das dificuldades para o diagnóstico e tratamento de doenças raras em nosso sistema público de saúde, o diagnóstico precoce de CLN2 precisa de melhorias pois a terapia de reposição enzimática está disponível e modifica o prognóstico dos pacientes.

## INTRODUCTION

Neuronal ceroid lipofuscinosis type 2 (CLN2) disease, formerly known as classical late-infantile neuronal ceroid lipofuscinosis or Jansky-Bielschowsky disease (OMIM#204500), is a neurodegenerative genetic disorder with an autosomal recessive inheritance. 1 2 Children with CLN2 have a deficiency of tripeptidyl peptidase 1 (TPP1), a soluble lysosomal enzyme, due to biallelic mutation in the *TPP1* gene, leading to abnormal deposition of lipopigments within the lysosomes and cell death, especially in neurons and in the retina. 1 2

Neuronal ceroid lipofuscinosis type 2 disease is rare among other metabolic diseases but not uncommon among genetic neurodegenerative disorders. 2 Epidemiological data are limited due to its rarity, and numbers from Brazil are not currently available. It is estimated that 14,000 people in the world have CLN2. 3 The global prevalence of CLN2 is 0.6–0.7 per million inhabitants. 3 In the classic form of CLN2, the first signs and symptoms occur in the early years of life, and children present progressive decline in language, cognitive and motor skills, drug-resistant epileptic seizures, visual loss, and premature death. 4 The enzyme replacement therapy (ERT) is available in the United States and European Union since 2017 and in Brazil since 2018. 5 6 7 It changes the course of the disease, with less language and motor function decline. 8

The diagnosis and management of CLN2 are challenging and need improvement. Most clinicians are unfamiliar with the disease, and initial symptoms are common in other neurological diseases. As a rare disease, evidence in CLN2 is scarce, and practical guidelines adapted to middle- to low-income countries with limited access to healthcare are not available. In this paper, we provide an expert consensus in the early diagnosis and management of CLN2 in Brazil, to help pediatricians and child neurologists to improve the outcomes of patients. The present original work shows the reality of our country regarding access to care and can help professionals from other countries with similar conditions.

## METHODS

The Brazilian Society of Child Neurology chose nine child neurologists, one of whom was also a medical geneticist, to compose the panel of experts. The selection was based on the clinical expertise of the professionals in CLN2, as they all work at referral centers for neuronal ceroid lipofuscinoses (NCLs) diseases and their familiarity with the limitations of the Brazilian healthcare system. The panelists were divided into 4 groups and elaborated 92 objective, clinically relevant multiple-choice questions concerning the diagnosis, the genetics, and the management of CLN2 in Brazil. This questionnaire was sent to each participant using an electronic platform (surveymonkey.com), where they answered individually and anonymously. An independent facilitator assembled the results and sent them to each participant. Based on the Delphi method, an answer chosen by at least 75% of the participants was considered a consensus. A virtual meeting was held to discuss the questions that did not reach a consensus. They performed the second round of voting using the same online tool. The disagreement was made explicit in this manuscript for the questions remaining without a consensus after this second round. The complete questionnaire with the results is available in Supplementary Material .

Each answer chosen by the expert panel was confronted with the best level of evidence (LE) found in the medical literature, and it was rated according to the 2009 Oxford Center for Evidence-Based Medicine Levels of Evidence classification. 9 The literature research was performed using the PubMed, Embase, and SciELO databases, with no date limitation, considering systematic reviews, randomized and nonrandomized clinical studies, observational studies, and case series, in English or Portuguese. Single case reports were excluded.

## CLINICAL MANIFESTATIONS

Neuronal ceroid lipofuscinose is a child neurodegenerative, autosomal recessive disease and represents the most common cause of dementia in the pediatric age group (consensus; LE: 2b). A cohort study including 2,636 children with dementia found that late-infantile NCL was the most frequent etiology among cases with confirmed diagnosis. 10 Another smaller cohort study reported a 2.1/100,000 prevalence of childhood dementia, and among the 80 identified cases, at least 7.5% had NCL as the leading cause. 11

Neuronal ceroid lipofuscinosis type 2 is a rapidly progressive type of NCL, and its classic form is the most prevalent phenotype (consensus; LE: 4), representing 87% of cases. 12 However, atypical cases with different phenotypes can be found; the largest cohort of atypical CLN2 from Latin America including 30 patients identified 7 cases from Brazil. 13 In classic CLN2, the onset of symptoms commonly occurs in early childhood, between 2 to 4 years old (consensus; LE: 2b). In a large cohort study with 140 cases, the median age of children with CLN2 was 2.9 years old for the first symptoms, taking on average 22.7 ± 9.8 months to diagnose after the first symptoms. 14 The mean age of death is 10 years old (consensus; LE: 2b), with a median time of 7.8 ± 0.9 years from diagnosis to death for patients without treatment. 14

The most common and early symptom of classic CLN2 is language delay (consensus; LE: 4), beginning at an early age, around 2 years old. 15 16 Patients may also develop normal language skills and present language regression around 3 years old. 16 Epileptic seizures are the second most common symptom (consensus; LE:4), appearing from 3 up to 6 years old, 15 16 and it is the main reason for parents to seek medical care (consensus; LE: 5). Myoclonic is the most typical seizure observed in patients with CLN2 disease (consensus; LE: 4), but tonic, tonic-clonic, and atonic seizure can also occur (consensus; LE: 2b). It is not unusual that patients present more than one type of seizure. 14 16 Epileptic seizures of any kind in a child with language delay are the “warning signs” for the clinical suspicion of CLN2 (consensus; LE: 2b), as they are the two most frequent early symptoms of CLN2. 14 Cerebellar ataxia and neuropsychomotor development regression are symptoms that, in addition to epilepsy, improve the pretesting suspicion index of CNL2 (consensus; LE: 5).

In the evolution of CLN2 disease, children have cognitive decline, ataxia, and progressive loss of vision (consensus; LE: 4), usually occurring within the 1 ^st^ year after the onset of language disability and seizures. 17 In a case series study evaluating 34 children with CLN2, 85% presented cognitive decline, 59% ataxia, and 59% impaired vision. 18 Other commons signs were involuntary movements, myoclonus, incoordination, pyramidal features, and abnormal behavior. 18 The severity of ophthalmic symptoms is closely related to the severity of neurological symptoms ( *p* < 0.03) and age ( *p* < 0.005), 19 (consensus; LE: 1c).

The atypical presentation of CLN2 corresponds to 13% of cases and has multiple phenotypes, including juvenile form (8% of cases), spinocerebellar ataxia or SCAR7 (3%), and spastic paraplegia or congenital form (< 1%) 12 (consensus; LE: 4). Children with atypical CLN2 have a later onset of symptoms, at around 6 years old, and the first symptom is epileptic seizures (consensus; LE: 4). In an evaluation of 30 atypical cases of CLN2 from South America, 47% presented seizures as the first symptom and the mean age of onset was 7.26 ± 3.84 years old, 20% language abnormality at 10.68 ± 8.53 years old, and 7% cognitive decline at 9.30 ± 7.57 years old. 13 The main symptoms that motivate parents to seek medical assistance are epileptic seizures, movement disorder, and ataxia (consensus; LE: 4), usually at 7 years old. 13 Around 41% of patients with atypical CLN2 have a second clinical manifestation within 1 year after the first symptom, usually when seizures were the first symptom, 13 but there is not a consensus in our panel of which symptom is most frequent. According to a case series with 14 patients with atypical CLN2, ataxia, language regression, seizures, and vision disturbance are the most common clinical manifestation with the progression of the disease. 20 However, only 3% of the South America cohort presented visual impairment in the first year; the mean age for the appearance of this symptom was 11.47 ± 5.04 years old. 13 Cognitive decline was present in 93% of the population of the present study, 7% had it as first symptom and 67% occurred within the 1 ^st^ year after the appearance of seizures. 13

## DIAGNOSIS

**Figure 1:**
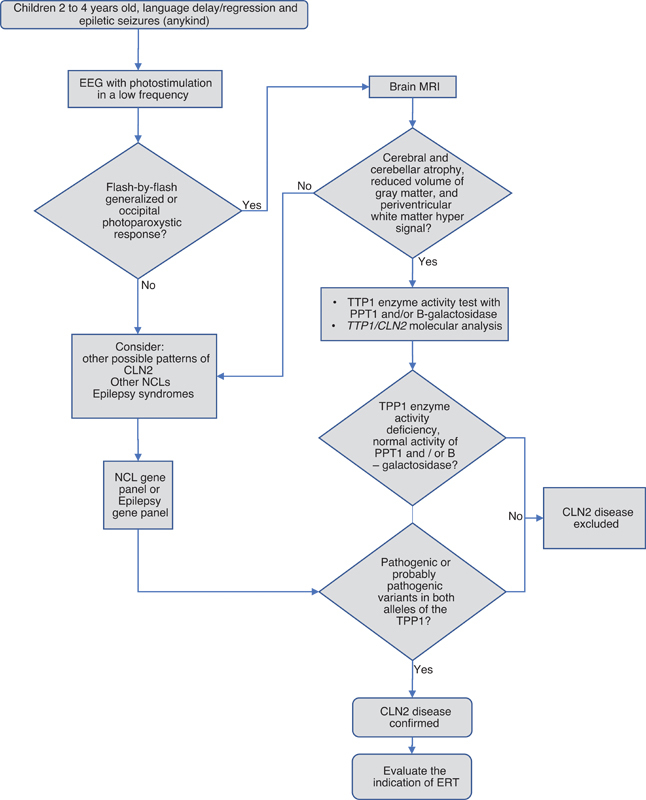
Abbreviations: CLN2, Neuronal ceroid lipofuscinosis type 2; EEG, electroencephalogram; ERT, enzyme replacement therapy; MRI, magnetic resonance imaging; NCL, Neuronal ceroid lipofuscinosis; TTP1, tripeptidyl peptidase 1; PTT1, palmitoyl protein thioesterase 1. Algorithm for diagnosis of CLN2.

An algorithm to help the diagnosis of CLN2 in Brazil in presented in Figure 1 .

### The EEG

**Figure 2:**
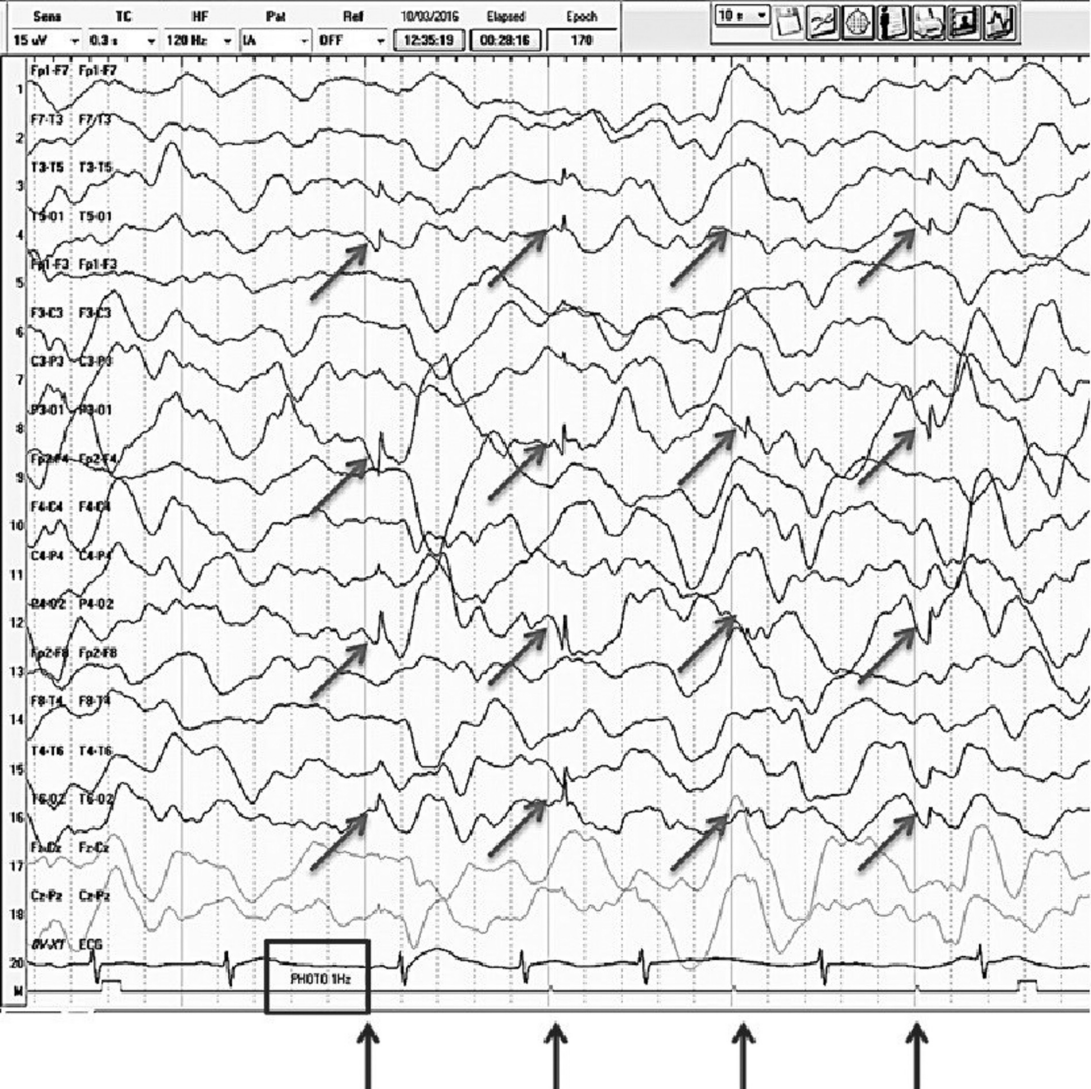
Electroencephalogram with slow frequency stimulation (1Hz) in a patient with CLN2. The EEG shows a flash-by-flash response characterized by occipital discharges with frequency identical to the stimulation. This is the most suggestive electroencephalographic pattern of CLN2.

The first complementary exams for a child between 2 and 4 years old, with language delay and the onset of epileptic seizures is an awake and asleep electroencephalogram (EEG), requested with photostimulation in a low frequency to assess the photoparoxystic response (consensus; LE: 4). The photoparoxystic response occurs at any frequency, but it is more evident at a low frequency. The most suggestive electroencephalographic pattern of CLN2 is a flash-by-flash generalized or occipital photoparoxystic response especially to low-frequency stimulus (1–2Hz) ( Figure 2 ) (consensus; LE: 2b). In other words, the EEG shows discharges with greater amplitude in the occipital regions in response especially to the low frequency photic stimulus, with frequency identical to the stimulation in the occipital region. 21 Even though other neurologic conditions may present photoparoxystic response in the EEG, the evaluation of 15 confirmed cases of CLN2 showed photoparoxystic response in 60% of the study population and, among them, 63% had bi-occipital or generalized spike waves in low-frequency stimulus. 22 In another cohort study, with 12 patients, 75% presented the photoparoxystic pattern, with an occipital spike-wave response. 23 In a Brazilian case series, all five patients with CLN2 presented photoparoxystic response especially in low frequency with posterior spikes. 24 In patients with CLN2, discharges especially appear at the lowest frequencies at photic stimulation (1, 2, and 3Hz) and the standard EEG is conducted with a frequency > 2 Hz; therefore, it is essential to demand evaluation of EEG in a low frequency of photic stimulation. These findings usually appear in the early phases and disappear as the disease progresses. 22 It is also essential to carry out an awake and sleep EEG because a characteristic of patients with CLN2 is the absence or scarcity of sleep spindles. 22 It is uncommon, but some patients may present abnormality only during sleep. 22 Other possible finding observed in the EEG are abnormal background activity, 24 generalized and multifocal epileptiform activity, 17 25 epileptiform activity in temporal regions, 23 epileptiform activity in posterior regions, 25 26 and photoparoxystic response to high-frequency stimulus (10 to 15Hz), 26 (consensus, LE: 4). The EEG is a simple exam but challenging to obtain in Brazil. The territorial extension of this country limits the access to a good quality EEG, as we have only few centers supported by universities with specialists in child neurology and neurophysiology.

### Neuroimaging

**Figure 3:**
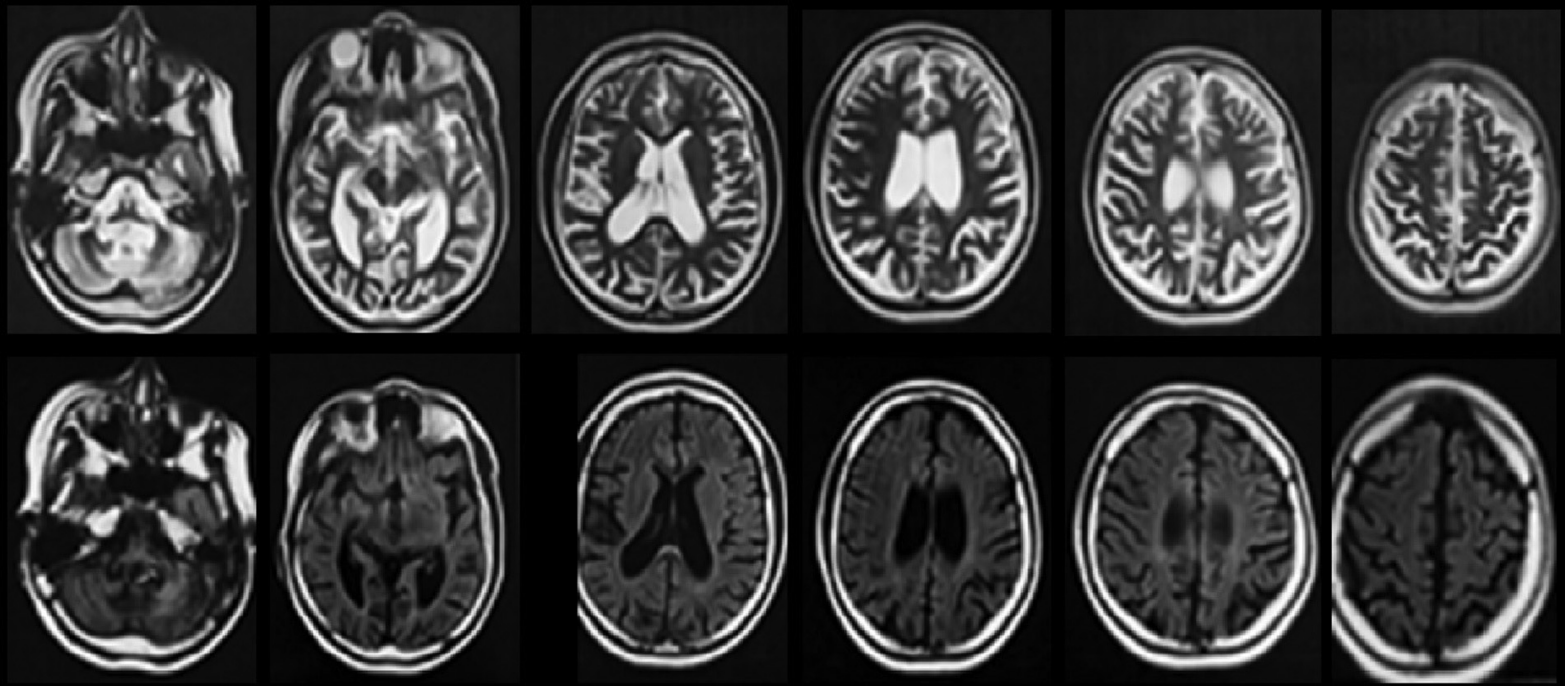
Brain MRI in a patient with CLN2 disease. Axial T2-weighted (first line) and FLAIR (second line) slices show cortical-subcortical atrophy with ex-vacuum ventricular dilatation and cerebellar atrophy.

The investigation should proceed with a brain magnetic resonance imaging (MRI) (consensus; LE: 5). The abnormalities observed in the brain MRI of patients with CLN2 are cerebral and cerebellar atrophy, reduced volume of gray matter, and periventricular white matter hyper signal ( Figure 3 ) (consensus; LE: 4). These findings are directly related to the severity of the disease. 16 In a case series including 12 patients, 92% had cerebellar atrophy, 67% cerebral atrophy, 83% linear hyperintensity of central white, 50% thinning of the corpus callosum, and 8% had thalamic hypointensity. 27 A Brazilian case series with 5 patients showed cerebral and cerebellar atrophy in 80% of patients. 24 The most common neuroimaging findings in patients with atypical CLN2 are cerebral and cerebellar atrophy and white matter abnormalities (consensus; LE: 4), presented in 87% of patients. 13

### Laboratory tests

In a child with a clinical presentation suggestive of CLN2, including neuropsychomotor regression, epilepsy, and ataxia, enzymatic and molecular tests should be requested, and there is no consensus in our panel concerning which molecular test should be performed first (either search for the pathogenic variant of the CLN2 gene or new generation sequencing/epileptic encephalopathies panel). The gold standard for the diagnosis of CLN2 is the detection of TPP1 enzyme activity deficiency with the normal activity of a control enzyme (consensus; LE: 1c) and the molecular analysis, with pathogenic or probably pathogenic variants in both alleles of the *TPP1* gene (consensus; LE: 4). The palmitoyl protein thioesterase 1 (PPT1) and / or B – galactosidase is used as control enzyme to exclude CLN1 if the enzymatic test is performed before molecular analysis or to ensure that the sample has not lost stability in case is done after the molecular analysis.

### Enzymatic analysis

The detection of TPP1 enzyme activity has high sensitivity and specificity for the diagnosis of CLN2, 28 and this test is available with the assistance of the pharmaceutical industry and the Hospital de Clínicas de Porto Alegre (Porto Alegre, RS, Brazil), where all the samples from Brazil are sent to be analyzed. The most recommended analysis for assessing the enzymatic activity of TPP1 is leukocyte analysis (consensus; LE: 1c), showing high sensitivity to detect any residual TPP1 activity. 28 Saliva and dry blood spot are also reliable methods. 29 30 In a cohort with 118 patients from Latin America initially selected by clinical assessment of NCL-compatible signs and symptoms, the molecular analysis identified 9 new cases of CLN2, with similar TPP-1 enzyme activity results obtained from leukocytes, saliva, and dry blood spot. 31

### Molecular analysis

The best strategy for genetic research of CLN2 is new generation sequencing (NGS) - Gene panel with copy number variation (CNV) analysis (consensus; LE: 1b). However, this exam is not available in the Brazilian public healthcare system; the pharmaceutical industry provides the financial support, and private health insurance may cover the costs, but prior request is needed. It is a challenge since both tests, enzymatic and molecular, are necessary to prescribe the enzymatic replacement treatment (ERT) of CLN2 (consensus; LE: 5).

The gene panel triples the yield of diagnosis in lysosomal storage diseases when comparing to biochemical test. 32 Once variants have been identified, the classification according to the American College of Medical Genetics and Genomics (ACMG) criteria (pathogenic, likely pathogenic, benign, likely benign, uncertain significance) 33 should be included in the report (consensus; LE: 5).

The molecular analysis with the epilepsy panel using NGS should be carried out in patients of 2 to 4 years old presenting language delay and epileptic seizures increasing in frequency despite treatment, even if the EEG has nonspecific findings and brain MRI is normal (consensus; LE: 5). The epilepsy panel covers genes for various etiologies of epilepsy, including the different forms of NCLs, bringing the possibility of an earlier diagnosis of CLN2. 34 In a cohort with 541 children presenting a first seizure from 2 years old, the NGS-based epilepsy panel diagnosed 21% of the population and identified 11 new cases of CLN2. 35 The NGS technique shows many advantages over the Sanger sequencing because it is time-saving and cost-effective, assessing multiple genes simultaneously. 36 37 When using the NGS, it is important to know whether exons and proximal intronic regions have been fully covered (consensus; LE: 5). It is also important to check if exonic deletions or duplications (also known as CNVs) were analyzed using NGS appropriate protocols, although this is not a common molecular mechanism in CLN2. 37

### Other complementary exams

The visual evoked potential (VEP) and the electroretinogram are not necessary to diagnose CLN2 (consensus; LE: 4), and the access to those exams is limited in Brazil. Visual impairment is not an early symptom in CLN2, and VEP may be absent. 17 38 When present, increased latency is the most common pattern in CLN2, present in up to 50% of patients. 17 38 An electroretinogram is challenging to carry out in children, and it is subject to errors from the examiner (consensus; LE: 5). In a case series evaluating the first symptoms of patients with CLN2 ( *n* = 9), only 3 presented retinopathy. 17 Nonetheless, a Brazilian case series showed that all five patients with CLN2 evaluated presented abnormal electroretinogram. 39

Electron microscopic analysis was widely used before the advance of molecular testing for diagnosis and classification of NCLs. Patients with CLN2 present curvilinear bodies as an intracellular accumulation pattern at the skin, conjunctive, and rectal biopsy observed under electron microscopy (consensus; LE: 4). It can also be found in lymphocytes and muscle. 38 Nowadays, a skin biopsy is no longer mandatory for the diagnosis (consensus; LE: 5). It could be helpful in areas with no access to molecular analysis, but it also requires expertise.

### Family study

There was not a consensus among our panelists, but parental genotyping is always indicated when the results between biochemistry and molecular are not conclusive, as long as the two variants identified are heterozygous, to ensure that the variants are on distinct alleles, that is, in trans. If the variants are homozygous, parental genotyping does not help the diagnosis.

In a family with a case of CLN2, testing should be extended for all siblings of the same age, for those younger, and for those who are older with little age difference, regardless of symptoms (consensus; LE: 5). Early, or presymptomatic diagnosis allows treatment to be initiated under optimal conditions. Thus, the investigation of younger siblings or even a little older is indicated, considering the possibility of discreet variation in the phenotype within the same family.

## MANAGEMENT

### General management

The therapeutic approach to CLN2 includes a multiprofessional team, emphasizing the functional maintenance and quality of life (consensus; LE: 5). Due to the broad spectrum of symptoms and the complexity of CLN2, the multidisciplinary team that manages these patients should include physicians (pediatric pulmonologist, pediatrician, child neurologist), professionals in physiotherapy, speech therapy, nutrition, occupational therapy, psychology, and social assistance (consensus; LE: 5).

Palliative care is the therapeutic approach in advanced stages, and the Brazilian public health system offers this intervention in more developed regions of the country. The important measures in palliative care are pain control, respiratory and hemodynamic support, and family assistance, targeting patients' quality of life and managing complications (consensus; LE: 5). Pain is frequent: parents of patients with CLN2 declare pain from once a week to every day; intensity is usually recognized as moderate to severe and disturbs sleep, mood, and daily activities. 40 The challenges in pain management increase as the deterioration of verbal communication advances.

The nutritional approach for patients with CLN2 should be composed of a balanced diet with adequate caloric intake, divided into smaller volumes (consensus; LE: 5). The main criteria for gastrostomy are dysphagia with episodes of recurrent respiratory infections or weight loss ≥ 10% of body weight in three months (consensus; LE: 4). In a case series report, 84 children with neurocognitive impairment received gastrostomy due to severe dysphagia (53% of the study population), malnutrition (21.4%), risk of aspiration pneumonia (10.7%), and aversion to food (7.1%). 41 After gastrostomy placement, the number of children presenting malnutrition decreased from 33.3% to 4.4%, *p* < 0.0001. 41 The frequency of respiratory infection also decreased significantly, *p* = 0.00037. 41

### Enzyme replacement therapy

Cerliponase α (Brineura, BioMarin Pharmaceutical Inc., Novato, CA, USA) is the specific treatment for CLN2 disease (consensus; LE: 1b). It is a recombinant human TTP-1 that cleaves the lysosomal tripeptides from the N-terminus of proteins (consensus; LE: 5), reducing the lysosomal material accumulation in the central nervous system (CNS). 42 Cerliponase α is commercially available in the United States and Europe since 2017 43 and in Brazil since 2018. 7 However, until the date of the present publication, the access to cerliponase α in Brazil is limited; neither the Brazilian public healthcare system nor the private health insurances provide the treatment.

The Hamburg scale is the most used scale in the clinical evaluation of patients with CLN2 ( Table 1 ) (consensus; LE: 5). It assesses the progression of CLN2 disease, evaluating the motor function, language, epileptic seizures, and visual function 44 (consensus; LE: 1b). A modified version, the Clinical Rating Scale, comprises only two domains (motor function and language) and is used in clinical studies to assess response to treatment 8 45 ( Table 1 ) (consensus; LE: 1b). Patients must present a score of at least three on the Clinical Rating Scale to be eligible for ERT.

**Table 1:** The Hamburg scale and Clinical Rating Scale used in the evaluation of CLN2 patients

	Score	Functional Level	
The Clinical Rating Scale	**Motor function**		The Hamburg Scale
	**3**	Walks normally;	
		No prominent ataxia, no pathologic falls	
	**2**	Frequent falls, obvious clumsiness;	
		But ability to walk without support for 10 steps	
	**1**	No unaided walking or crawling only	
	**0**	Immobile, mostly bedridden	
	**Language**			
	**3**	Normal;	
		Intelligible and grossly age-appropriate;	
		No decline noted yet	
	**2**	Has become recognizably abnormal	
	**1**	Hardly understandable;	
		Few intelligible words	
	**0**	Unintelligible or no language	
	**Seizures**			
	**3**	No seizure per 3-mo period	
	**2**	1 to 2 seizures per 3-mo period	
	**1**	1 seizure per month	
	**0**	> 1 seizure per month	
	**Visual function**			
	**3**	Recognizes desirable object, grabs at it	
	**2**	Grabbing for objects uncoordinated	
	**1**	Reacts to light	
	**0**	No reaction to visual stimuli	

Adapted from Steinfeld et al. 2002, and Schulz et al. 2018.

An open-label, multicenter, controlled study evaluated cerliponase α in 23 children with CLN2, aged from 3 to 16 years old, with a combined score of 3 to 6 on the Clinical Rating Scale. 8 Patients treated with Cerliponase α showed less motor and language decline than controls (HR: 0.08; 95% confidence interval [CI]: 0.02–0.23; *p* < 0.001), lower motor score alone (HR: 0.04; 95%CI: 0.00–0.29; *p* = 0.002), and lower language score alone (HR: 0.15; 95%CI: 0.04–0.52; *p* = 0.003). 8 Treated patients had a slower decline than controls: only 9% of the treated patients had a 2-point decline in 49.3 weeks versus 100% of controls. 8 Cerliponase α also controls epileptic seizures (consensus; LE: 1b), reducing their frequency. 8 15 46 Safety data from the clinical study and the ongoing extension trial showed that the incidence of seizures decreased from 88% to 26% in 24 weeks. 8 46 A case series including 13 patients from Australia reported no generalized tonic-clonic seizures after 1 year of treatment. 15

The recommended dose for treating individuals with CLN2 is 300 mg every 2 weeks, administered via intracerebroventricular (ICV) infusion, performed with a dilution of 300 mg in 10 ml and infused in 4 hours 8 (consensus; LE: 1b). The essential devices for the proper application are a permanent ICV infusion catheter (Ommaya type) and an infusion pump for a syringe (consensus; LE: 5). A Rickham ICV reservoir can also be used, 47 and they are both available in Brazil. A child neurologist, a neurosurgeon, a pediatrician, and a nurse should be involved in the team for the infusion (consensus; LE: 5). The minimum necessary conditions for the use of ERT to reduce the risk of infection or other complications are: (a) a room with isolation and strict asepsis; (b) a restriction in the number of people in the room during the infusion, avoiding visitors and other professionals not essential to the procedure; (d) regular monitoring of vital signs; and (e) monitoring with equipment throughout the infusion period (consensus; LE: 4). The infusion can be conducted in the hospital ward, as long as there are no other patients with infection (consensus; LE: 4). After the placement of the infusion catheter, immediate care should avoid contamination of the dressing and trauma at the procedure site (consensus; LE: 4). Reports from the literature show a low rate of device-related complications in patients with CLN2. 8 47 A retrospective evaluation of 48 patients with CLN2 receiving more than 3,000 sessions of ERT through ICV device reported an infection rate of 0.33 and 0.27% of non-infectious adverse events. 47 In the clinical trial with cerliponase α, 8% of the study population had an infection related to the device. 8 In this case, the best approach is to temporarily suspend the ERT administration, begin antibiotic treatment, and change the device (consensus; LE: 1b). Strict asepsis and an experienced team decrease the risk of complications of the implementation of the ICV device. The incidence of infection was only 1% in a cohort with 98 children with malignant brain tumors receiving a total of 5,472 sessions of intrathecal chemotherapy through Ommaya catheter. 48 The placement of the ICV device followed aseptic techniques and other precautions established as a protocol and had only trained staff to assist the procedure and to administer the drug. 48

One of the possible complications with the chronic treatment of ERT is obstruction or leakage of the system by repeated use (consensus; LE: 1b). In the cerliponase α clinical trial, 8% of children had device leakage and 4% had occlusion. 8 In a systematic review of ICV used for cancer treatment ( *n* = 620 children), 2.7% of the population presented CSF leakage and 0.8% obstruction of the device. 49 The best approach in those cases is to change the device (consensus; LE: 5).

The minimum period between the placement of the ventricular intracerebral device by the neurosurgeon until the first ERT infusion is 5 to 7 days or maybe longer, depending on local edema (consensus; LE: 5). The ICV catheter must be changed every 4 years to decrease the infection or CSF leakage risk (consensus; LE: 5). However, it is important to highlight that the ICV catheter has been used for more than 50 years in pediatric oncology and presents a good long-term safety profile. 50 In a cohort evaluating 1,515 children with CNS tumors, 96% of children showed no complication 10 years after the ICV placement. 50

The eligible patients with CLN2 for ERT are children, ≥ 3 years old, with a Clinical Rating Scale ≥ 3 points (consensus; LE: 1b). There is no current data to support the beneficial effect in patients with a score below three, and the benefits in a more advanced stage of the disease are not clear (consensus; LE: 5). We could consider the possibility of slowing down the disease, but there is insufficient scientific data to state (consensus; LE: 5). Patients from a French real-world cohort study presenting scores < 3 at the beginning of ERT did not show any improvement of motor function or language disability. 51 Children with a confirmed diagnosis of pre-symptomatic CLN2 should be treated with cerliponase α (consensus; LE: 5). However, there is not enough evidence to define the best time to start treatment in those patients as the clinical trial only evaluated symptomatic patients 8 (consensus; LE: 5). In the French real-world cohort with eight patients treated with cerliponase, only one patient received pre-symptomatic treatment at 1 year old, but longer follow-up is needed to evaluate the benefits. 51

To minimize the side effects of cerliponase α, we should infuse an antihistamine drug 30 minutes before the enzyme (consensus; LE: 1b). Fever is the most frequent side effect after infusion (consensus; LE: 1b), occurring in 71% of children. 8 In this case, antipyretics and temperature maps are indicated (consensus; LE: 5). Headache, irritability, vomiting, skin rash (topical dermatitis, urticaria); epileptic seizures may also occur after cerliponase α infusion (consensus; LE: 1b), and treatment is also symptomatic (consensus; LE: 5). During the clinical trial, 96% of patients presented seizures, but only 6% were considered treatment-related. 8 Sixty-three percent presented vomiting, 63% hypersensitivity reactions, 54% upper respiratory tract infections, 42% nasopharyngitis, and 42% rhinitis. 8

There is no need to collect cerebrospinal fluid (CSF) in every infusion of cerliponase α (consensus; LE: 5). The presence of pleocytosis and elevated protein in the CSF must be analyzed in the clinical context of the patient because it does not always indicate CNS infection (consensus; LE: 1b). In the study with 23 children, 17% presented abnormalities in the CSF (increased white cell count) without evidence of infection. 8 We should not routinely investigate the presence of antibodies related to medication in CSF, and their presence is not related to the severity of the disease (consensus; LE: 1b). The results from phase I and II studies evaluating the immunogenicity of cerliponase α after 129 weeks of treatment showed no association between the presence of anti-drug antibody and hypersensitivity adverse events. 52 The presence of antibodies was not related to the motor and language score. 52

There is not enough data to establish the criteria for discontinuing the use of cerliponase α (consensus; LE: 5).

### Antiepileptic drugs

Regarding the rational use of antiepileptic drugs in patients with CLN2, a slow and gradual titration is recommended, giving preference for monotherapy and low doses to decrease the risk of adverse events (consensus; LE: 5). However, most patients end up on polytherapy, as the disease is progressive and poorly responsive to anti-seizure drugs. In this pathology, due to its progressive character, we seek a clinical improvement of the epilepsy, as we will hardly achieve the desired goal: the normalization of the EEG and/or the complete resolution of the seizures. The most recommended drugs as first-line therapy are valproate, clobazam, clonazepam, and levetiracetam (consensus; LE: 5).

Carbamazepine should be avoided, as it can worsen myoclonic seizures in patients with CLN2 (consensus; LE: 4). The evaluation of 28 patients receiving carbamazepine to treat myoclonic epilepsy, 68% presented aggravation, and only 14% showed a positive response. 53

In cases of status epilepticus, the use of phenobarbital and midazolam are therapeutic options (consensus, LE; 5), available in most public services in our country. Phenytoin should be avoided in patients with CLN2 presenting acute seizures in emergency services (consensus; LE: 5), because it may worsen myoclonic seizures. 53

### Approach to other clinical manifestations

The medications of choice for spasticity are baclofen and botulinum toxin (consensus; LE: 5). Baclofen treats generalized spasticity, 54 55 and a high dose is usually necessary for patients with CLN2 due to the intensity of spasticity. Botulinum toxin reduces local spasticity and improves muscle tone, 56 and it is available in the Brazilian public healthcare. Benzodiazepines should not be used as long-term treatment as they increase respiratory secretion, muscle weakness, and ataxia. 54

Movement disorders, such as myoclonus, ataxia, dystonia, parkinsonism, and chorea, are frequent in patients with NCLs 57 and have been described in patients with CLN2. 16 58 According to our experts, the drug used for myoclonus is levetiracetam and for parkinsonism is dopamine; trihexyphenidyl and biperiden are indicated for dystonia with no preference among them (consensus; LE: 5).

More than 90% of patients with CLN2 present sleep disorders. 59 Melatonin helps regulate the sleep and wake cycle, especially in patients with impaired vision, 60 and non-pharmacological measures, such as sleep hygiene, could also be beneficial (consensus; LE: 5).

Behavioral disorders may be one of the initial symptoms of CLN2 and affects up to 16% of patients. 14 The best strategy to manage is identifying and treating trigger factors, behavioral approach, parental training, and symptomatic medication (consensus; LE: 5). Risperidone is the best therapeutic option for behavioral disorders associated with CLN2 (consensus; LE: 5).

Anxiety and depression are directly related to the stage of the disease and the use of ERT. 61 In a child with signs suggestive of depression, the drugs available for treatment are fluoxetine, fluvoxamine, or sertraline, with no preference among them (consensus; LE: 5).

In conclusion, CLN2 is a rare and not easily recognizable disease. Initial manifestations are not specific to CLN2, as other inborn errors of metabolism and spinocerebellar ataxias may have a similar clinical presentation, delaying the suspicion. The approval of ERT has completely changed the management of CNL2 patients, highlighting the importance of early diagnosis to allow treatment initiation.

Expert opinions and guidelines in CLN2 are found in the medical literature, but none considering the difficulties in low- and middle-income countries. We identified reports of CLN2 cases from Brazil, but no recommendation for the diagnosis and management focused on the limited access to care in the country.

In the present paper, we compiled our medical expertise, the findings from the literature, and the challenges of the healthcare system in Brazil. Most statements in the present paper were ranked as low level since most evidence comes from case series. Nevertheless, this consensus has a significant value in practice because the rarity of the disease with the paucity of clinical trials justified our findings.
