# Supplementary material for: Clinical management and diagnosis of CLN2 disease: consensus of the Brazilian experts group
Source: Arq Neuropsiquiatr. 2023 Apr 14;81(3):284–95. doi: 10.1055/s-0043-1761434 (PMC10104757; doi:10.1055/s-0043-1761434)
Supplement: Supplementary file 1 — Supplementary Material [file 10-1055-s-0043-1761434-s210391.pdf]

SUPPLEMENTARY MATERIAL

Questionnaire and results

I. Clinical manifestations

01. In CLN2 disease, the most common and early symptom is:	
a) Axial ataxia	0%
**b) Language delay**	**88.89%**
c) Epileptic seizures	11.11%
d) Involution of neurodevelopment	0%
02. To increase the level of clinical suspicion of CLN2, what another symptom follows the most common and early one described above?	
**a) Epileptic seizures**	**88.89%**
b) Language delay	11.11%
c) Blindness	0%
d) Involution of neurodevelopment	0%
03. In the evolution of CLN2 disease, children may have:	
a) Cognitive decline	0%
b) Ataxia	0%
c) Progressive loss of vision	0%
**d) All alternatives are correct**	**100%**
04. In CLN2 disease, which symptom most leads parents to seek medical attention?	
a) Blindness	0%
b) Dystonia	0%
**c) Epileptic seizures**	**100%**
d) Language regression	0%
05. What is the typical epileptic seizure that occurs in CLN2?	
a) Feverish	0%
b) Generalized tonic-clonic	0%
c) Absence	0%
**d) Myoclonic**	**100%**
06. In CLN2, the severity of ophthalmic symptoms is closely related to:	
a) Severity of neurological symptom	0%
b) Age	22.22%
c) Severity of neurological symptoms and age	55.56%
d) Regardless of the severity of neurological symptoms and age	22.22%
06re-voted. In CLN2, the severity of ophthalmic symptoms is closely related to:	
a) Severity of neurological symptom	0%
b) Age	0%
**c) Severity of neurological symptoms and age**	**100%**
d) Regardless of the severity of neurological symptoms and age	0%
07. What do we consider "warning signs" for the clinical suspicion of CLN2?	
a) Ataxia and dystonia	0%
**b) Epileptic seizures of any kind in children with language delay**	**88.89%**

 © 2023. Academia Brasileira de Neurologia. All rights reserved.

(*Continued*)

c) Blindness and dystonia	0%
d) Ataxia and blindness	11.11%
08. CLN2 is a rapidly progressing pediatric neurodegenerative disease whose onset of symptoms commonly occurs in the age group between:	
a) Newborn to 2 years	0%
**b) 2 - 4 years**	**100%**
c) 4 - 6 years	0%
d) 6 - 8 years	0%
09. Cases of atypical presentation of CLN2 can present with multiple phenotypes, including:	
a) Progressive dystonia	11.11%
**b) Autosomal recessive spinocerebellar ataxia type 7 (SCAR7)**	**88.89%**
c) GLUT1 deficiency	0%
d) Vitamin B12 deficiency	0%
10. Patients with atypical presentation of CLN2 commonly present, as first symptoms:	
a) Epileptic seizures	22.22%
b) Language disorder	0%
c) Behavioral disorder	0%
**d) All of the above are correct**	**77.78%**
11. The age of onset of the atypical presentation of CLN2 disease is:	
a) Earlier, around 12 months	0%
b) Later, around 8 years	33.33%
c) Later, most commonly around 6 years	66.67%
d) Between 2 - 4 years	0%
11re-voted. The age of onset of the atypical presentation of CLN2 disease is:	
a) Earlier, around 12 months	0%
b) Later, around 8 years	0%
**c) Later, most commonly around 6 years**	**100%**
d) Between 2 - 4 years	0%
12. What other type of crisis can the patient with CLN2 present?	
a) Tonic	0%
b) Tonic-clonic	0%
c) Atonic	0%
**d) All types of crises described above can occur in CLN2**	**100%**
13. The average age of death of the patient with CLN2 is usually around:	
a) 4 years old	0%
b) 5 years old	0%
c) 6 years old	11.11%
**d) 10 years old**	**88.89%**
14. The classification of neuronal lipofuscinosis is based on the age of onset of symptoms, the most common form is:	
a) Congenital: onset of symptoms near birth	0%
b) Infant: onset of symptoms around 5 months to 18 months	0%
**c) Late infantile: onset of symptoms around 2-4 years**	**100%**
d) Juvenile onset of symptoms over 5 to 10 years	0%

(*Continued*)

 © 2023. Academia Brasileira de Neurologia. All rights reserved.

(*Continued*)

15. Among the atypical forms of CLN2 some phenotypes were described clinically as:	
a) CLN juvenile form	0%
b) Spinocerebellar ataxia or SCAR7	11.11%
c) Spastic paraplegia and congenital form	0%
**d) All of the above are correct**	**88.89%**
16. Neuronal ceroid lipofuscinosis is a pediatric neurodegenerative disease, autosomal recessive, and represents the most common cause of:	
a) Ataxia in the pediatric age group	0%
b) Dystonia in the pediatric age group	0%
c) Epilepsy in the pediatric age group	11.11%
**d) Dementia in the pediatric age group**	**88.89%**
17. In atypical CLN2, the main symptoms that motivate parents to seek medical assistance are:	
a) Epileptic seizures	11.11%
b) Movement disorder	11.11%
c) Ataxia	0%
**d) All of the above**	**77.78%**
18. The most common neuroimaging findings in patients with atypical CLN2 most commonly described are:	
a) Cerebellar atrophy	22.22%
b) Changes in white matter	0%
c) Cerebral atrophy	0%
**d) All of the above**	**77.78%**
19. To assess the progression of CLN2 disease, the University Medical Center Hamburg-Eppendorf has developed a scale, which assesses the following domains:	
**a) Motor function, epileptic seizures, visual function and language**	**100%**
b) Motor and visual function only	0%
c) Eating and swallowing	0%
d) Visual function and language	0%
20. We can state about the clinical assessment scale adapted for CLN2 disease:	
a) It is a modified version of the Hamburg-Eppendorf scale	0%
b) It comprises only 2 domains: motor function and language	11.11%
c) It is the scale used in clinical studies to assess response to the treatment of CLN2	11.11%
**d) All of the above are correct**	**77.78%**
21. In atypical forms of CLN2, around 41% of patients have a second clinical manifestation within one year after the first symptom. All of the statements below are correct, except:	
a) The epileptic seizure is the first symptom	0%
b) Cognitive decline is the main symptom that arises after the first symptom of the disease	55.56%
c) Difficulties in language are also frequent after the appearance of the first symptom	0%
d) Visual changes are frequent at this time of the disease	44.44%
21re-voted. In atypical forms of CLN2, around 41% of patients have a second clinical manifestation within one year after the first symptom. All of the statements below are correct, except:	
a) The epileptic seizure is the first symptom	0%
b) Cognitive decline is the main symptom that arises after the first symptom of the disease	33.33%
c) Difficulties in language are also frequent after the appearance of the first symptom	0%
d) Visual changes are frequent at this time of the disease	66.67%

 © 2023. Academia Brasileira de Neurologia. All rights reserved.

II. Diagnosis

22. In a child between 2 and 4 years of age, with language delay and onset of epileptic seizures, which test should I order initially for diagnosis?	
**a) EEG - request EEG while awake and asleep, with slow photo stimulation to assess the photoparoxystic response**	**100%**
b) Hearing assessment	0%
c) Magnetic resonance imaging of the skull	0%
d) Magnetic resonance with spectroscopy	0%
e) Test for detection of TPP1 enzyme activity	0%
f) Molecular analysis - search for the pathogenic variant of the CLN2 gene	0%
g) Molecular analysis - new generation sequencing / epilepsy panel	0%
h) Electron microscopy: skin or lymphocyte biopsy	0%
23. What changes can be observed in the EEG of patients with CLN2?	
a) Disorganization of the basic activity	0%
b) Generalized and multifocal epileptiform activity	0%
c) Epileptiform activity in temporal regions	0%
d) Epileptiform activity in posterior regions	0%
e) Photoparoxystic response to the high frequency stimulus (10-15Hz)	0%
f) Photoparoxystic response with low frequency stimulus (1-2Hz):	33.33%
g) All alternatives are correct	66.67%
23re-voted. What changes can be observed in the EEG of patients with CLN2?	
a) Disorganization of the basic activity	0%
b) Generalized and multifocal epileptiform activity	0%
c) Epileptiform activity in temporal regions	0%
d) Epileptiform activity in posterior regions	0%
e) Photoparoxystic response to the high frequency stimulus (10-15Hz)	0%
f) Photoparoxystic response with low frequency stimulus (1-2Hz)	0%
**g) All alternatives are correct**	**100%**
24. What is the most suggestive electroencephalographic pattern of CLN2?	
a) Generalized photoparoxystic response to the high frequency stimulus (10-15Hz)	0%
b) Photoparoxystic response in the brain regions after the high frequency stimulus (10-15Hz)	0%
**c) Generalized photoparoxystic response or in the occipital region to low frequency stimulus (1-2Hz)**	**100%**
25. How to proceed with the investigation? Which second exam should I request for the diagnostic evaluation of this child?	
a) EEG - request EEG while awake and asleep, with slow photo stimulation to assess the photoparoxystic response	0%
b) Hearing assessment	0%
**c) Magnetic resonance imaging of the skull**	**88.89%**
d) Magnetic resonance with spectroscopy	11.11%
e) Test for detection of TPP1 enzyme activity	0%
f) Molecular analysis - search for the pathogenic variant of the CLN2 gene	0%
g) Molecular analysis - new generation sequencing / epilepsy panel	0%
h) Electron microscopy: skin or lymphocyte biopsy	0%
26. What changes can be observed in the Magnetic Resonance Imaging of patients with CLN2?	
a) Cerebellar atrophy	0%
b) Increase in the peak of NAA in spectroscopy	0%
c) Cerebral atrophy	0%
d) Bridged hypersignal and midbrain	0%

(*Continued*)

 © 2023. Academia Brasileira de Neurologia. All rights reserved.

(*Continued*)

e) Reduction in the volume of gray matter	0%
f) Periventricular white matter hypersignal	0%
**g) All alternatives are correct, except (b) and (d)**	**100%**
27.This child, aged 2 to 4 years, with language delay and epileptic seizures increasing in frequency despite treatment, has EEG with nonspecific findings and Magnetic Resonance Imaging of the normal skull. Which exam should I request at this time for diagnostic investigation?	
a) EEG - request EEG while awake and asleep, with slow photo stimulation to assess the photoparoxystic response	11.11%
b) Hearing assessment	0%
c) Magnetic resonance imaging of the skull	0%
d) Magnetic resonance with spectroscopy	0%
e) Test for detection of TPP1 enzyme activity	11.11%
f) Molecular analysis - search for the pathogenic variant of the CLN2 gene	0%
**g) Molecular analysis - new generation sequencing / epilepsy panel**	**77.78%**
h) Electron microscopy: skin or lymphocyte biopsy	0%
28. Upon returning with the exams, the neurologist observes that the child also has ataxia, leading him to suspect CLN2 or another progressive disease, which exam should he order?	
a) EEG - request EEG while awake and asleep, with slow photo stimulation to assess the photoparoxystic response.	0%
b) Hearing assessment	0%
c) Magnetic resonance imaging of the skull	0%
d) Magnetic resonance with spectroscopy	0%
e) Test for detection of TPP1 enzyme activity	11.11%
f) Molecular analysis - search for the pathogenic variant of the CLN2 gene	11.11%
g) Molecular analysis - new generation sequencing / epileptic encephalopathies panel	11.11%
h) Electroretinogram	0%
i) Visual evoked potential	0%
j) Electron microscopy: skin or lymphocyte biopsy	0%
l) Options (e) and (g) or (f)	66.67%
28re-voted. Upon returning with the exams, the neurologist observes that the child also has ataxia, leading him to suspect CLN2 or another progressive disease, which exam should he order?	
a) EEG - request EEG while awake and asleep, with slow photo stimulation to assess the photoparoxystic response.	0%
b) Hearing assessment	0%
c) Magnetic resonance imaging of the skull	0%
d) Magnetic resonance with spectroscopy	0%
e) Test for detection of TPP1 enzyme activity	0%
f) Molecular analysis - search for the pathogenic variant of the CLN2 gene	0%
g) Molecular analysis - new generation sequencing / epileptic encephalopathies panel	55.56%
h) Electroretinogram	0%
i) Visual evoked potential	0%
j) Electron microscopy: skin or lymphocyte biopsy	0%
l) Options (e) and (g) or (f)	44.44%
29. Which exams are considered the gold standard in the diagnosis of CLN2?	
a) EEG in wakefulness and sleep and MRI of the skull	0%
**b) Detection of TPP1 enzyme activity deficiency (together with normal activity of a control enzyme such as PPT1 and / or B - galactosidase) and molecular analysis - pathogenic variant in both alleles of the TPP1 / CLN2 gene**	**100%**
c) Electroretinogram and visual evoked potential	0%

 © 2023. Academia Brasileira de Neurologia. All rights reserved.

(*Continued*)

30. For the diagnosis of CLN2, do I need to request an electroretinogram?	
a) Yes	0%
b) It is not necessary, difficult to perform in children, subject to errors on the part of the examiner	55.56%
c) Only if no enzymatic and / or molecular analysis is available	11.11%
d) Only in the follow-up	22.22%
e) Only mention that it may be altered	11.11%
30re-voted. For the diagnosis of CLN2, do I need to request an electroretinogram?	
a) Yes	0%
**b) It is not necessary, difficult to perform in children, subject to errors on the part of the examiner**	**100%**
c) Only if no enzymatic and / or molecular analysis is available	0%
d) Only in the follow-up	0%
e) Only mention that it may be altered	0%
31. For the diagnosis of CLN2, do I need to request visual evoked potential?	
a) Yes	0%
**b) It is not necessary**	**77.78%**
c) Only if no enzymatic and / or molecular analysis is available	0%
d) Only in the follow-up	11.11%
e) Only mention that it may be altered	11.11%
32. For the diagnosis of CLN2, do I need to request a skin biopsy evaluated by electron microscopy?	
a) Yes	0%
b) No	33.33%
c) Only if enzymatic and / or molecular analysis is not available	66.67%
d) Only in the follow-up	0%
32re-voted. For the diagnosis of CLN2, do I need to request a skin biopsy evaluated by electron microscopy?	
a) Yes	0%
**b) No**	**100%**
c) Only if enzymatic and / or molecular analysis is not available	0%
d) Only in the follow-up	0%
33. To indicate the enzymatic treatment of CLN2, which tests do I need to have performed?	
a) EEG in wakefulness and sleep and MRI of the skull	0%
**b) Detection of TPP1 enzyme activity deficiency (together with normal activity of a control enzyme such as PPT1 and / or B - galactosidase) and molecular analysis - pathogenic variant in both alleles of the TPP1 / CLN2 gene**	**100%**
c) Electroretinogram and visual evoked potential	0%
34. What change can be observed in the Visual Evoked Potential of patients with CLN2?	
a) Decreased latency	22.22%
b) Increased latency	55.56%
c) Decreased amplitude	11.11%
d) Increased amplitude	11.11%
34re-voted. What change can be observed in the Visual Evoked Potential of patients with CLN2?	
a) Decreased latency	0%
**b) Increased latency**	**88.89%**
c) Decreased amplitude	11.11%
d) Increased amplitude	0%

(*Continued*)

 © 2023. Academia Brasileira de Neurologia. All rights reserved.

(*Continued*)

35. What is the most recommended analysis for assessing the enzymatic activity of TPP1?	
a) Leukocyte analysis	66.67%
b) Whole blood analysis	0%
c) Analysis of fibroblasts	22.22%
d) Analysis in dry blood	11.11%
e) Saliva analysis	0%
35re-voted. What is the most recommended analysis for assessing the enzymatic activity of TPP1?	
**a) Leukocyte analysis**	**100%**
b) Whole blood analysis	0%
c) Analysis of fibroblasts	0%
d) Analysis in dry blood	0%
e) Saliva analysis	0%
36. What is the expected intracellular accumulation pattern at skin / conjunctive / rectal biopsy observed under electron microscopy in patients with CLN2?	
**a) Curvilinear bodies**	**88.89%**
b) Inclusions in digital printing	11.11%
c) Retinear inclusions	0%
d) Osmophilic granular deposits	0%

III. Genetics

37. What is the best strategy for genetic research of CLN2?	
a) Sequencing by the Sanger method	0%
b) New generation sequencing - TPP1 only	0%
**c) New generation sequencing - Gene panel with copy number variation analysis**	**100%**
d) MLPA for CLN2	0%
38. What is important to know when using new generation sequencing?	
a) Whether exons and proximal intronic regions have been fully covered	66.67%
b) If each base had a reading equal to or greater than 20 readings	22.22%
c) If a search for variation in the number of copies was carried out by NGS	11.11%
38re-voted. What is important to know when using new generation sequencing?	
**a) Whether exons and proximal intronic regions have been fully covered**	**100%**
b) If each base had a reading equal to or greater than 20 readings	0%
c) If a search for variation in the number of copies was carried out by NGS	0%
39. Once variants have been identified, what should be included in the report?	
a) If the variant has been previously described and under what circumstances. Whether its repercussion on enzyme activity has been proven	0%
b) What the predictors of pathogenicity say	0%
c) In case of two heterozygous variants, they have been shown to be trans	11.11%
**d) What is the classification of the variants: VUS, probably pathogenic and pathogenic, according to the ACMG criteria**	**88.89%**
40. Should an altered genetic test always be confirmed by a biochemical study?	
a) It is desirable, but it is not necessary	0%
b) In case of homozygous variants proving to be pathogenic, it is not necessary	33.33%
c) In case of variants never described before, it is mandatory	66.67%

 © 2023. Academia Brasileira de Neurologia. All rights reserved.

(*Continued*)

d) B and C are correct	0%
40re-voted. Should an altered genetic test always be confirmed by a biochemical study?	
a) It is desirable, but it is not necessary.	0%
b) In case of homozygous variants proving to be pathogenic, it is not necessary	0%
c) In case of variants never described before, it is mandatory with copy number variation analysis	22.22%
**d) B and C are correct**	**77.78%**
41. The segregation study (parental genotyping) of the variant identified in the index case:	
a) It must always be done	22.22%
b) It must be carried out in special cases, in which the enzymatic activity has not been conclusive	22.22%
c) It does not need to be carried out, if the enzymatic activity is unequivocally altered	55.56%
41re-voted. The segregation study (parental genotyping) of the variant identified in the index case:	
a) It must always be done	0%
b) It must be carried out in special cases, in which the enzymatic activity has not been conclusive	55.56%
c) It does not need to be carried out, if the enzymatic activity is unequivocally altered	44.44%
42. If a case of CLN2 is identified in the family, testing should be extended to:	
a) All siblings, regardless of age, since there is intra-family variability in the phenotype	44.44%
b) Only for siblings with the same age, younger siblings, and for those older with little age difference, regardless of having symptoms	44.44%
c) Only for those who have symptoms	11.11%
42re-voted. If a case of CLN2 is identified in the family, testing should be extended to	
a) All siblings, regardless of age, since there is intra-family variability in the phenotype	0%
**b) Only for siblings with the same age, younger siblings, and for those older with little age difference, regardless of having symptoms**	**100%**
c) Only for those who have symptoms	0%
43. When genetic research by NGS finds variants only in heterozygosity or homozygosity in VUS, which further investigation would be more appropriate to confirm the diagnosis?	
a) Enzymatic assays of TPP1 in leukocytes	44.44%
b) Sanger sequencing for the possibility of intronic mutation	0%
c) Sanger sequencing or enzymatic assays if the clinic is compatible	55.56%
43re-voted. When genetic research by NGS finds variants only in heterozygosity or homozygosity in VUS, which further investigation would be more appropriate to confirm the diagnosis?	
**a) Enzymatic assays of TPP1 in leukocytes**	**100%**
b) Sanger sequencing for the possibility of intronic mutation	0%
c) Sanger sequencing or enzymatic assays if the clinic is compatible	0%
44. In the development of a genetic panel for CLN2, which clinical syndrome should serve as an "entry" in addition to epilepsy in order to increase the pre-testing suspicion index?	
a) Cerebellar ataxia	66.67%
b) Dystonia	0%
c) Visual loss	0%
d) Neuropsychomotor development regression	33.33%
e) Alternatives A and D are correct	0%
44re-voted. In the development of a genetic panel for CLN2, which clinical syndrome should serve as an "entry" in addition to epilepsy in order to increase the pre-testing suspicion index?	
a) Cerebellar ataxia	0%
b) Dystonia	0%
c) Visual loss	0%
d) Neuropsychomotor development regression	0%

(*Continued*)

 © 2023. Academia Brasileira de Neurologia. All rights reserved.

IV. Treatment

45. Concerning prenatal diagnosis or neonatal screening: Should children with a confirmed diagnosis of pre-symptomatic CLN2 start treatment with Cerliponase alfa?	
**a) Yes**	**88.89%**
b) No	11.11%
46. What are the criteria for starting treatment with Cerliponase alfa in children diagnosed pre-symptomatic?	
a) Immediate, before any clinical manifestation or changes in complementary exams such as EEG or MRI of the brain	66.67%
b) If there is a change in the EEG photo-stimulation, before the clinical manifestations	0%
c) If you show signs of cerebral or cerebellar atrophy on neuroimaging, before clinical manifestations	0%
d) When speech delay or regression is identified	33.33%
e) There is not enough evidence to support the decision	0%
46re-voted. What are the criteria for starting treatment with Cerliponase alfa in children diagnosed pre-symptomatic?	
a) Immediate, before any clinical manifestation or changes in complementary exams such as EEG or MRI of the brain	0%
b) If there is a change in the EEG photo-stimulation, before the clinical manifestations	0%
c) If you show signs of cerebral or cerebellar atrophy on neuroimaging, before clinical manifestations	0%
d) When speech delay or regression is identified	0%
**e) There is not enough evidence to support the decision**	**100%**
47. Which first-line antiepileptic drugs are used to control epileptic seizures in patients with CLN2?	
a) Carbamazepine, phenytoin, phenobarbital and primidone	0%
**b) Valproate, clobazam, clonazepam, levetiracetam**	**100%**
c) Topiramate, phenobarbital, oxcarbazepine, ethosuximide	0%
d) Lamotrigine, valproate, primidone, vigabatrin	0%
48. Which drug should be avoided, as it can worsen myoclonic seizures in patients with CLN2?	
a) Valproate	0%
b) Clobazam	0%
c) Phenobarbital	0%
**d) Carbamazepine**	**100%**
49. Regarding the rational use of antiepileptic drugs in patients with CLN2, it is recommended:	
a) Drug polytherapy and high doses early	33.33%
b) Slow and gradual titration, preference for monotherapy, low doses	66.67%
c) Drugs with predominant action on focal crises	0%
d) Quickly try various drugs to achieve total seizure control	0%
49re-voted. Regarding the rational use of antiepileptic drugs in patients with CLN2, it is recommended:	
a) Drug polytherapy and high doses early	0%
b) Slow and gradual titration, preference for monotherapy, low doses	**100%**
c) Drugs with predominant action on focal crises	0%
d) Quickly try various drugs to achieve total seizure control	0%
50. What drug should be avoided in situations of acute epileptic seizures in emergency services?	
**a) Phenytoin**	**77.78%**
b) Phenobarbital	0%
c) Benzodiazepine	22.22%
51. What are the therapeutic options for status epilepticus in emergency services?	
**a) Phenobarbital and Midazolam**	**100%**
b) Phenobarbital and Phenytoin	0%
c) Phenytoin and Benzodiazepine	0%

 © 2023. Academia Brasileira de Neurologia. All rights reserved.

V. Support treatment and multidisciplinary team

52. Which professionals should compose the multidisciplinary team for the support management of patients with CLN2?	
a) Physiotherapist and occupational therapist, as progressive motor changes are the hallmark of the disease	0%
b) Doctors of various specialties	0%
c) There is rarely a need for speech therapy, because dysphagia is late symptom	0%
**d) Doctors (pneumologist, pediatrician, neuropediatrician) and professionals in physiotherapy, speech therapy, nutrition, occupational therapy, psychology, social assistance, among others**	**100%**
53. What are the main goals in the supportive therapeutic approach for patients with CLN2?	
a) Multiprofessional rehabilitation, aiming to reverse the progressive loss of psychomotor acquisitions	0%
b) The rehabilitation must be focused and focused on the patient, as the full recovery of this patient will benefit the whole family	0%
**c) Multiprofessional approach focused on the patient and family, with an emphasis on functional maintenance and quality of life for the patient and family**	**100%**
d) Use of medications to treat disease complications	0%
54. Supportive treatment involving patient and family should be composed of which approaches?	
**a) Quality of life, multiprofessional approach, palliative care in the advanced stages of the disease and family support and support**	**100%**
b) Intensive rehabilitation, medication management, early permanent hospitalization	0%
c) Early drug therapy, palliative care in advanced stages of the disease, parenteral nutrition	0%
d) Surgical approach to treat deformities, drug therapy, use of orthoses and auxiliary means of locomotion	0%
55. Which nutritional dietary approach is appropriate for patients with CLN2?	
a) Hypoprotein diet to prevent diarrhea	0%
**b) Balanced diet with adequate caloric intake, divided into smaller volumes**	**100%**
c) Diet and milk formula specific to CLN2 disease	0%
d) Liquid diet since diagnosis for better absorption	0%
56. What are the main criteria for gastrostomy indication during the course of the disease?	
**a) Dysphagia with episodes of recurrent respiratory infections or weight loss equal to or greater than 10% of weight in 3 months**	**100%**
b) Dysphagia only	0%
c) Episodes of recurrent respiratory infection	0%
57. What are the medications of choice for the treatment of spasticity?	
**a) Baclofen and Botulinum toxin**	**77.78%**
b) Baclofen and physiotherapy	11.11%
c) Baclofen and benzodiazepine	11.11%
58. What is the therapeutic option for movement disorders, such as parkinsonism?	
a) Dopamine	44.44%
b) Triexiphenidyl	22.22%
c) Biperiden	33.33%
d) There is no preference among the drugs mentioned above	0%
58re-voted. What is the therapeutic option for movement disorders, such as parkinsonism?	
a) Dopamine	0%
b) Triexiphenidyl	0%
c) Biperiden	0%
**d) There is no preference among the drugs mentioned above**	**100%**
59. For sleep disorders in patients with CLN2, preferably:	
a) Long half-life benzodiazepines	11.11%
b) Typical neuroleptics	0%

(*Continued*)

 © 2023. Academia Brasileira de Neurologia. All rights reserved.

(*Continued*)

**c) Melatonin, for helping to regulate the sleep and wake cycle taking into account the impaired vision of patients, and non-pharmacological measures, such as sleep hygiene**	**88.89%**
d) Tricyclic antidepressants	0%
60. What better approach to behavioral disorders?	
**a) Identify and treat trigger factors, behavioral approach, parental training and symptomatic medication**	**100%**
b) Early use of typical neuroleptics	0%
c) Use of long-acting benzodiazepines	0%
d) Tricyclic antidepressants and MAO inhibitors	0%
61. What is the best therapeutic option for behavioral disorders associated with CLN2?	
**a) Risperidone**	**100%**
b) Aripripazole	0%
c) Neozine	0%
d) Haloperidol	0%
62. Child with signs suggestive of depression, what is the therapeutic option?	
a) Fluoxetine / Fluvoxamine	44.44%
b) Sertraline	55.56%
c) Quetiapine	0%
d) There is no preference among the drugs mentioned above	0%
62re-voted. Child with signs suggestive of depression, what is the therapeutic option?	
a) Fluoxetine / Fluvoxamine	0%
b) Sertraline	0%
c) Quetiapine	0%
**d) There is no preference among the drugs mentioned above**	**100%**

VI. Enzyme replacement therapy (ERT)

63. What specific treatment for CLN2 has been commercially available so far?	
a) Antisense oligonucleotide	0%
**b) ERT (Cerliponase alfa)**	**100%**
c) Gene therapy	0%
d) Substrate Reduction Therapy	0%
64. Which devices are essential for a proper application of Cerliponase alfa?	
**a) Permanent intracerebroventricular infusion catheter (Ommaya type) and infusion pump for syringe**	**100%**
b) Port-a-cath and infusion pump	0%
c) Ommaya catheter and cardiac monitoring	0%
65. Which professionals should be involved in the team for the infusion of Cerliponase alfa?	
a) Pediatrician, neuropediatrician and nurse	0%
b) Psychologist, pediatrician and neuropediatrician	0%
**c) Neuropediatrician, neurosurgeon, pediatrician and nurse**	**100%**
66. What is the immediate care after the placement of the infusion catheter (Ommaya)?	
a) To avoid contamination of the dressing and trauma at the procedure site	55.56%
b) To avoid contamination of the dressing	22.22%
c) To avoid trauma at the procedure site, which may lead to rupture of the device or displacement of the same	22.22%

 © 2023. Academia Brasileira de Neurologia. All rights reserved.

(*Continued*)

66re-voted. What is the immediate care after the placement of the infusion catheter (Ommaya)?	
**a) To avoid contamination of the dressing and trauma at the procedure site**	**100%**
b) To avoid contamination of the dressing	0%
c) To avoid trauma at the procedure site, which may lead to rupture of the device or displacement of the same	0%
67. What is Brineura®'s infusion route?	
**a) Intracerebral ventricular**	**100%**
b) Intravenous	0%
c) Intrathecal	0%
68. Identify the minimum conditions necessary for the use of ERT:	
a) Room with isolation and strict asepsis	66.67%
b) Avoid visitors and any other not essential professionals from the hospital during the infusion	33.33%
c) The infusion can be performed in a hospital ward, as long as there are no other patients with infection	22.22%
d) Regular vital signs monitoring by professional	33.33%
e) Monitoring with equipment throughout the infusion period	11.11%
f) All alternatives above	0%
68re-voted. Identify the minimum conditions necessary for the use of ERT:	
a) Room with isolation and strict asepsis	0%
b) Avoid visitors and any other not essential professionals from the hospital during the infusion	0%
c) The infusion can be performed in a hospital ward, as long as there are no other patients with infection	0%
d) Regular vital signs monitoring by professional	0%
e) Monitoring with equipment throughout the infusion period	0%
**f) All alternatives above**	**100%**
69. What is the minimum period between the neurosurgeon's placement of the ventricular intracerebral device until the first ERT infusion?	
a) 15 days	33.33%
b) 5 to 7 days, or may be longer depending on local edema	44.44%
c) 1 week, no more than that	22.22%
d) 30-45 days after the surgical procedure	0%
69re-voted. What is the minimum period between the neurosurgeon's placement of the ventricular intracerebral device until the first ERT infusion?	
a) 15 days	0%
**b) 5 to 7 days, or may be longer depending on local edema**	**100%**
c) 1 week, no more than that	0%
d) 30-45 days after the surgical procedure	0%
70. Is there a need to change the intracerebroventricular catheter?	
a) No, the catheter lasts for life	11.11%
b) Yes, and the suggested period is every 4 years	66.67%
c) Yes, and the appropriate period is every 10 years	22.22%
70re-voted. Is there a need to change the intracerebroventricular catheter?	
a) No, the catheter lasts for life	0%
**b) Yes, and the suggested period is every 4 years**	**100%**
c) Yes, and the appropriate period is every 10 years	0%

(*Continued*)

 © 2023. Academia Brasileira de Neurologia. All rights reserved.

(*Continued*)

71. Which scale is most used in the clinical evaluation of patients with CLN2?	
**a) Hamburg scale**	**100%**
b) SARA (Scale for the Assessment and Rating of Ataxia)	0%
c) Weill Cornell Scale	0%
72. In the clinical trial of individuals with CLN2, what was the cutoff point on the Hamburg scale used for the use of Brineura®?	
a) There was no cutoff point	0%
**b) ≥3 points**	**100%**
c) ≥5 points	0%
73. Is there a beneficial effect of Brineura® medication if it starts in a phase of the disease with a score below 3 on the Hamburg scale?	
a) Yes, there are data proving	33.33%
b) There is no additional benefit	0%
c) Possibly, considering that the progression of the disease is associated with the accumulation of non-metabolized lipofuscin	66.67%
d) There is no currently available data to support this issue	0%
73re-voted. Is there a beneficial effect of Brineura medication if it starts in a phase of the disease with a score below 3 on the Hamburg scale?	
a) Yes, there are data proving	0%
b) There is no additional benefit	0%
c) Possibly, considering that the progression of the disease is associated with the accumulation of non-metabolized lipofuscin	0%
**d) There is no currently available data to support this issue**	**100%**
74. Would a patient at any more advanced stage of the disease have benefits with the use of Brineura®?	
a) Yes, there are case reports proving	0%
b) There are no benefits, as there will be no regression of symptoms	11.11%
**c) There is a possibility of slowing down the disease, but there is insufficient scientific data to state**	**88.89%**
75. To minimize the side effects of Brineura®, which medication should be infused before the enzyme?	
**a) Antihistamine 30 minutes before**	**88.89%**
b) Corticoid 1 hour before	11.11%
c) Immunoglobulin 24 hours before	0%
76. What is the pharmacodynamics of Cerliponase alfa?	
**a) Lysosomal cleavage of tripeptides**	**88.89%**
b) Interruption in lysosomal tripeptide traffic	11.11%
77. What dose of Cerliponase alfa is recommended for the treatment of individuals with CLN2?	
**a) 300 mg, every two weeks**	**100%**
b) 300 mg weekly	0%
c) 300 mg / kg / dose, weekly	0%
78. How should Cerliponase alfa infusion be carried out?	
a) Dilution of 300 mg in 10 ml and infuse in 4 hours	55.56%
b) Dilution of 300 mg in 100 ml of 0.9% SF and infuse in 4 hours	44.44%
c) Dilution of 300 mg in 500 ml of 0.9% SF and infuse in 12 hours	0%
78re-voted. How should Cerliponase alfa infusion be carried out?	
**a) Dilution of 300 mg in 10 ml and infuse in 4 hours**	**100%**
b) Dilution of 300 mg in 100 ml of 0.9% SF and infuse in 4 hours	0%
c) Dilution of 300 mg in 500 ml of 0.9% SF and infuse in 12 hours	0%

 © 2023. Academia Brasileira de Neurologia. All rights reserved.

(*Continued*)

79. What is the most frequent side effect after infusion?	
a) Fever	66.67%
b) Vomiting	22.22%
c) Headache	11.11%
79re-voted. What is the most frequent side effect after infusion?	
**a) Fever**	**100%**
b) Vomiting	0%
c) Headache	0%
80. What should be done with a child presenting fever after infusion of ERT?	
a) Use of antipyretic and temperature map	44.44%
b) Infectious screening	55.56%
c) Antihistamine	0%
80re-voted. What should be done with a child presenting fever after infusion of ERT?	
**a) Use of antipyretic and temperature map**	**100%**
b) Infectious screening	0%
c) Antihistamine	0%
81. Does ERT with Cerliponase alfa interfere in the control of epileptic seizures?	
a) Yes, it can help to reduce the frequency of crises	66.67%
b) It does not interfere	22.22%
c) Yes, it can worsen epileptic seizures	11.11%
81re-voted. Does ERT with cerliponase alfa interfere in the control of epileptic seizures?	
**a) Yes, it can help to reduce the frequency of crises**	**100%**
b) It does not interfere	
c) Yes, it can worsen epileptic seizures	
82. Indicate other clinical manifestations, in addition to fever, that may occur after Brineura® infusion?	
**a) Headache, irritability, vomiting, skin rash (topical dermatitis, urticaria), epileptic seizures**	**100%**
b) Somnolence, headache, diarrhea	0%
c) Diplopia, skin rash (topical dermatitis, urticaria), edema of limbs	0%
83. What is the conduct regarding the signs and symptoms mentioned above?	
**a) Use of symptomatic**	**77.78%**
b) Hospitalization	0%
c) Suspension of medication infusion	22.22%
84. Is there a need to collect Cerebrospinal fluid (CSF) every time you are going to infuse the medication?	
a) Yes	44.44%
b) No	55.56%
84re-voted. Is there a need to collect CSF every time you are going to infuse the medication?	
a) Yes	11.11%
**b) No**	**88.89%**
85. Is the presence of pleocytosis and elevated protein in the CSF indicative of central nervous system infection?	
a) Yes always	0%
b) Never	0%
**c) It must be analyzed in the clinical context of the patient**	**100%**
86. Should we routinely investigate the presence of antibodies related to medication in CSF?	
a) Yes	33.33%
b) No	66.67%

(*Continued*)

 © 2023. Academia Brasileira de Neurologia. All rights reserved.

(*Continued*)

86re-voted. Should we routinely investigate the presence of antibodies related to medication in CSF?	
a) Yes	0%
**b) No**	**100%**
87. Could the presence of anti-cerliponase alpha antibodies in the CSF be related to the severity of the disease?	
a) Yes	22.22%
**b) No**	**77.78%**
88. In the case of the presence of leakage or rupture of the infusion device for Cerliponase alfa, what is the best approach?	
**a) Changing the device**	**77.78%**
b) Suspension of medication	11.11%
c) Interruption of medication	11.11%
89. In case of central nervous system infection related to the device, what is the best approach?	
**a) Temporarily suspend the enzyme, prescribe antibiotic treatment and change the device**	**77.78%**
b) Use of antibiotics and replacement of the device	11.11%
c) Maintain medication and associate antibiotics with corticosteroids	11.11%
90. What are the complications that can arise with the chronic treatment of ERT?	
a) None, as it is a very secure system	0%
b) Obstruction or leakage of the system by repeated use	66.67%
c) Fibrosis around the system, in the region below the scalp, making access to the catheter difficult	0%
d) There is insufficient information on the prolonged use of this catheter	33.33%
90. What are the complications that can arise with the chronic treatment of ERT?	
a) None, as it is a very secure system	0%
**b) Obstruction or leakage of the system by repeated use**	**100%**
c) Fibrosis around the system, in the region below the scalp, making access to the catheter difficult	0%
d) There is insufficient information on the prolonged use of this catheter	0%

VII. Palliative care

91. Which palliative measures are important in advanced stages of the disease?	
a) Even patients in advanced and severe stages should receive Cerliponase Alpha	0%
b) Polytherapy with antiepileptic drugs for total seizure control	0%
**c) Pain control measures, respiratory and hemodynamic support, family support**	**100%**
d) Hypothermia and mannitol as neuroprotection	0%
92. When to discontinue Brineura® medication?	
a) When the disease progresses and reaches the score = 0 on the Hamburg scale	62.5%
b) When the disease progresses with a loss of 1 point on the Hamburg scale, in relation to the score at the beginning of treatment	37.5%
c) Medication should not be suspended until the patient's death	0%
d) There is not enough data to support this decision	0%
92re-voted. When to discontinue Brineura® medication?	
a) When the disease progresses and reaches the score = 0 on the Hamburg scale 62.5%	0%
b) When the disease progresses with a loss of 1 point on the Hamburg scale, in relation to the score at the beginning of treatment 37.5%	0%
c) Medication should not be suspended until the patient's death	0%
**d) There is not enough data to support this decision**	**100%**

 © 2023. Academia Brasileira de Neurologia. All rights reserved.
